# RNA-Seq Analyses Reveal Roles of the HVCN1 Proton Channel in Cardiac pH Homeostasis

**DOI:** 10.3389/fcell.2022.860502

**Published:** 2022-03-16

**Authors:** Xin Wu, Yawei Li, Mark Maienschein-Cline, Leonid Feferman, Longjun Wu, Liang Hong

**Affiliations:** ^1^ Department of Medicine, University of Illinois at Chicago, Chicago, IL, United States; ^2^ Department of Preventive Medicine, Northwestern University, Chicago, IL, United States; ^3^ Research Informatics Core, Research Resources Center, University of Illinois at Chicago, Chicago, IL, United States; ^4^ Department of Neurology, Mayo Clinic, Rochester, MN, United States

**Keywords:** HVCN1, RNA-seq, NOX, pH homeostasis, heart

## Abstract

The voltage-gated proton channel HVCN1 is a member of the voltage-gated ion channel family. HVCN1 channel controls acid extrusion and regulates pH homeostasis in various cell types. Recent evidence indicated that the HVCN1 channel was associated with cardiac function. To investigate the role of HVCN1 in cardiac myocytes, we performed an RNA sequencing analysis of murine hearts and showed that HVCN1 null hearts exhibited a differential transcriptome profile compared with wild-type hearts. The RNA-seq data indicating impaired pH homeostasis in HVCN1 null hearts were the downregulated NADPH oxidoreductases (NOXs) and decreased expression of Cl^−^/HCO_3_^−^ exchanger, indicating HVCN1 is a regulator of gene transcriptional networks controlling NOX signaling and CO_2_ homeostasis in the heart. Additionally, HVCN1 null hearts exhibited differential expression of cardiac ion channels, suggesting a potential role of HVCN1 in cardiac electrophysiological remodeling. The study highlights the importance of HVCN1 in cardiac function and may present a novel target associated with heart diseases.

## Introduction

The voltage-gated proton channel HVCN1 is a member of the voltage-gated ion channel family (Ramsey et al., 2006; Sasaki et al., 2006). It is composed of two subunits. Each subunit contains a proton-permeable voltage-sensing domain and lacks the pore domain typical of other voltage-gated ion channels. The HVCN1 proton channel is highly selective for H^+^ (Berger and Isacoff, 2011; Musset et al., 2011), and plays a crucial role in regulating pH homeostasis in various cell types (DeCoursey, 2013).

The HVCN1 channel extrudes protons during the respiratory burst of the NADPH oxidoreductases (NOXs) in phagocytes. It provides charge and pH compensation and controls the production of reactive oxygen species (ROS) and H_2_O_2_ by NOX (Seredenina et al., 2015). In the nervous system, HVCN1 is required for NOX-dependent ROS/H_2_O_2_ generation in brain microglia in the central nervous system (Wu et al., 2012; Peng et al., 2021). HVCN1 is a sperm flagellar regulator of intracellular pH and plays a crucial role in sperm capacitation (Lishko et al., 2010). It mediates H^+^ efflux at the pulmonary alveolar cell membrane and acidifies excessively alkaline airway surface liquid in the airway cells (Iovannisci et al., 2010). In addition, HVCN1 activity is required for acid extrusion to shape action potentials in snail neurons (Thomas and Meech, 1982), and contributes to efficient proton efflux in algal cells to sustain intracellular calcification in coccolithophores (Taylor et al., 2011). We previously developed guanidine derivatives as HVCN1 inhibitors (Hong et al., 2013; Hong et al., 2014; Zhao et al., 2021a; Zhao et al., 2021b). Pharmacologic inhibition of HVCN1 activity by these blockers has been reported to alter sperm motility (Matamoros-Volante and Trevino, 2020; Yeste et al., 2020), promote leukemic Jurkat T cell apoptosis (Asuaje et al., 2017), and inhibit breast cancer progression (Ventura et al., 2020).

Recently, accumulating evidence indicated that the HVCN1 channel was involved in cardiac function. A previous study reported that a voltage-activated proton current in human cardiac fibroblasts regulated intracellular pH and membrane potential (El Chemaly et al., 2006). Another study showed that the change of cardiac pH_i_ was voltage-sensitive (Yamamoto et al., 2005). Recent transcriptome analyses indicated that the voltage-gated proton channel HVCN1 mRNAs were expressed in the heart at high levels comparable to Cl^−^/HCO_3_^−^ exchangers (Brawand et al., 2011; Yu et al., 2014; Vairamani et al., 2017). These studies suggest a potential role of voltage-gated proton channel HVCN1 in cardiac pH homeostasis.

To explore the effects of HVCN1 gene deletion on cardiac function, we performed an RNA-profiling analysis of HVCN1 null and wild-type mouse hearts. The HVCN1^−/−^ hearts showed a significantly differential transcriptome profile. The deletion of HVCN1 altered the expression of cardiac NADPH oxidoreductases, bicarbonate transporter, and ion channels associated with the cardiac electrophysiological profile.

## Materials and Methods

### Animals

The experimental procedures in mice and the protocol used in this study were approved by the University of Illinois at Chicago (UIC) Animal Care Committee (ACC No. 19-178). All animal studies were performed according to approved guidelines for the use and care of live animals, and conformed with animal care policies and procedures of UIC. The HVCN1^−/−^ and wild-type (C57BL/6 background) mice were used in the study. Mice were housed in temperature- and humidity-controlled rooms with 12-h light-dark cycles in the animal care facility at the UIC. We studied male mice, similarly aged HVCN1^−/−^ mice (n = 4, 3 months old) and wild-type control mice (n = 4, 3 months old) were euthanized and heart samples were isolated for analysis.

### RNA-Seq Analysis

The RNA-seq analysis was performed as previously described (Hong et al., 2021). We first extracted RNA with Maxwell^®^ RSC simplyRNA Cells Kit (Promega AS1390) based on manufacturer’s instructions, using Maxwell^®^ RSC Instrument (Promega AS4500), and then RNA was quantified using Qubit 4.0 Fluorometer (Invitrogen) with the Qubit RNA HS Assay Kit (REF Q32855) analyzed for integrity using the Agilent 4200 TapeStation RNA ScreenTape assay (Agilent 5067–5576, 5067–5577). RNA samples were normalized to 250ng, and library prep was carried out using the universal Plus mRNA-Seq kit (NuGen 0520-A01) as written in the product manual (NuGen M01485 v5). In brief, RNA underwent poly-A selection, enzymatic fragmentation, and generation of double-stranded cDNA using a mixture of Oligo (dT) and random priming. The cDNA underwent end repair, ligation of dual-index adaptors, strand selection, and 15 cycles of PCR amplification. We determined the number of cycles by qPCR of a small aliquot of un-amplified libraries. All intermediate purification steps, and final library purification were carried out using Agencourt AMPure XP Beads (Beckman Coulter A63881). We measured purified library concentrations with the Qubit 1X dsDNA HS Assay Kit (Invitrogen Q33231), and fragment size distribution was confirmed using the D5000 ScreenTape assay (Agilent 5067–5588, 5067–5589), and libraries were pooled in equimolar amounts based on the Qubit concentration and TapeStation average size and run on MiniSeq for index balancing. The libraries were re-pooled with corrected inputs based on the % Reads Identified (PF) results from the MiniSeq run, and the new pool was purified with the Agencourt AMPure XP Beads (Beckman Coulter A63881). The final, purified pool was quantified by qPCR using the KAPA Library Quantification Kit and run on a NovaSeq6000 SP flow cell, 2 × 50 nt, one lane, at the University of Illinois Roy J. Carver Biotechnology Center High-Throughput Sequencing and Genotyping Unit. Raw reads were aligned to the reference genome hg38 using STAR (Dobin et al., 2013). ENSEMBL gene expression was quantified using FeatureCounts (Liao et al., 2014). Normalized and differential expression statistics were computed using edgeR (Robinson et al., 2010; McCarthy et al., 2012), and *p*-values were adjusted for multiple testing using the false discovery rate (FDR) correction of Benjamini and Hochberg. We performed unsupervised hierarchical clustering of all differentially expressed genes using the Euclidean distance and complete linkage method, and generated volcano plots using R. Up- and down-regulated genes were analyzed separately using the DAVID functional annotation tool and the Gene Ontology Biological Process (GO BP) database. Bar charts of the top 20 most significant upregulated and downregulated pathways, based on FDR-corrected *p*-value, were constructed with the ggplot2 R package. We constructed heatmaps for the cardiac conduction and calcium ion transport into cytosol pathways with the ComplexHeatmap R package.

### Quantitative Real-Time PCR (RT-PCR) Analysis

RT-PCR analysis was performed as previously described (Kim et al., 2014). Total RNA was isolated from hearts of adult wild-type or HVCN1^−/−^ mice using Trizol RNA extraction agent (Invitrogen) according to the manufacturer’s instructions. For the reverse transcription, total RNA was quantified using the Nanodrop spectrophotometer (Thermofisher Scientific). Synthesis of cDNA was carried out with SuperScript IV RNase Reverse transcriptase (Invitrogen) and primers with 2 μg of total RNA as template. Real-time PCR was performed on an Applied Biosystems (Thermofisher Scientific) to determine mRNA levels of differentially expressed genes according to the manufacturer’s recommended protocol using SYBR green assays. For each group, data were collected from three independent samples; three replicas were performed for each sample. The primer sequences used for SYBR green-based fluorescence were *GAPDH*: 5′-AGG​TCG​GTG​TGA​ACG​GAT​TTG-3′ and 5′-TGT​AGA​CCA​TGT​AGT​TGA​GGT​CA-3′; *NOX1*: 5′-TTC​CTC​ACT​GGC​TGG​GAT​AG-3′ and 5′-AGT​CCG​AGG​GCC​ACA​TAA​GA-3′; *NOX2*: 5′-TGG​CGA​TCT​CAG​CAA​AAG​GTG​G-3′ and 5′-GTA​CTG​TCC​CAC​CTC​CAT​CTT​G-3′; *NOX4*: 5′-TCT​GGA​AAA​CCT​TCC​TGC​TG-3′ and 5′-CCG​GCA​CAT​AGG​TAA​AAG​GA-3′; *SLC4A1*: 5′-CCG​TGA​ACT​CTT​CAT​TGC​TGC​C-3′ and 5′-ACC​AGG​AAC​AGC​AAG​CTC​ATG​C-3′; *SLC4A2*: 5′-GCA​CCT​CCA​TTC​TGT​TTG​CGG​T-3′ and 5′-GCT​CAC​GAA​CTT​CCA​ACA​CAG​C-3′; *SLC4A3*: 5′-GGT​GCT​GAT​TGC​CTT​CTC​CAG​T-3′ and 5′-GAC​AAC​GAA​GCC​AGA​GGA​GAA​G-3′; *SLC26A6*: 5′-TAC​CGT​GTG​GAC​AGT​AAC​CAG​G-3′ and 5′-CCT​GTA​CCA​AGC​TCC​GAG​ACA​T-3′.

### Data and Statistical Analysis

All data were presented as the mean ± SEM. The normality in each group was determined by the *Shapiro-Wilk test*, and *p > 0.05* was considered to indicate normally distributed. Homoscedasticity was determined by the *two-sample F test*. For equal variances between two groups, significance between means was determined by *Student’s t-test*. For unequal variances between groups, significance between means was determined by *Welch’s t-test. p < 0.05* was considered to indicate a statistically significant difference.

## Results

### RNA Sequencing Analysis Revealed a Differential Transcriptome Profile in HVCN1^−/−^ Mouse Hearts

**FIGURE 1:**
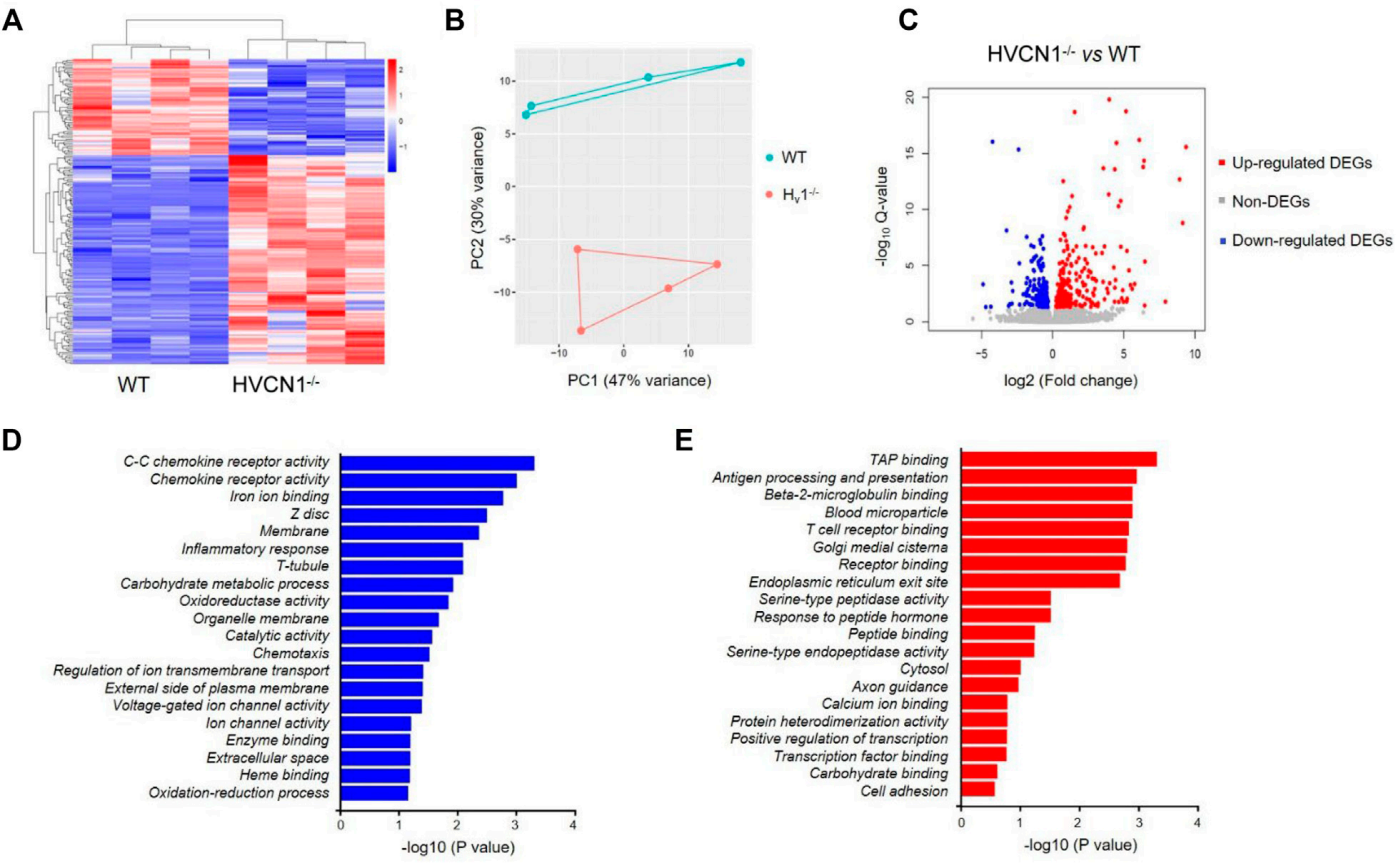
RNA sequencing analysis revealed a differential transcriptome profile in HVCN1^−/−^ mouse heart. **(A)** Heatmap and hierarchical clustering of DEGs in the wild-type and HVCN1^−/−^ hearts, n = 4 hearts for each group. **(B)** Principal component analysis of wild-type and HVCN1^−/−^ hearts. **(C)** The volcano plot has red points representing upregulated DEGs, blue representing downregulated DEGs, and gray points representing non-DEGs. **(D-E)** Gene Ontology enrichment analysis of top significant gene groups either downregulated **(D)** or upregulated **(E)**.

To explore the potential function of HVCN1 in the heart, we performed a transcriptomic analysis of wild-type (WT) and HVCN1^−/−^ mouse hearts. The HVCN1 null hearts exhibited a significantly different transcriptome profile compared with WT hearts (Figure 1 and Supplementary Table S1). Hierarchical clustering of differentially expressed genes (DEGs) in WT and HVCN1^−/−^ hearts showed two main clusters with samples of the same group clustered together (Figures 1A,B). A total of 206 DEGs were identified with 67 downregulated and 139 upregulated (Figure 1C). The major Gene Ontology (GO) categories identified by RNA sequencing and the most significantly modulated in the HVCN1^−/−^ mouse hearts included immunological activity, ion and receptor binding, metabolic process, and ion channel activity (Figures 1D,E).

### Downregulation of the NOX Family of ROS-Generating NADPH Oxidases in HVCN1^−/−^ Hearts

**FIGURE 2:**
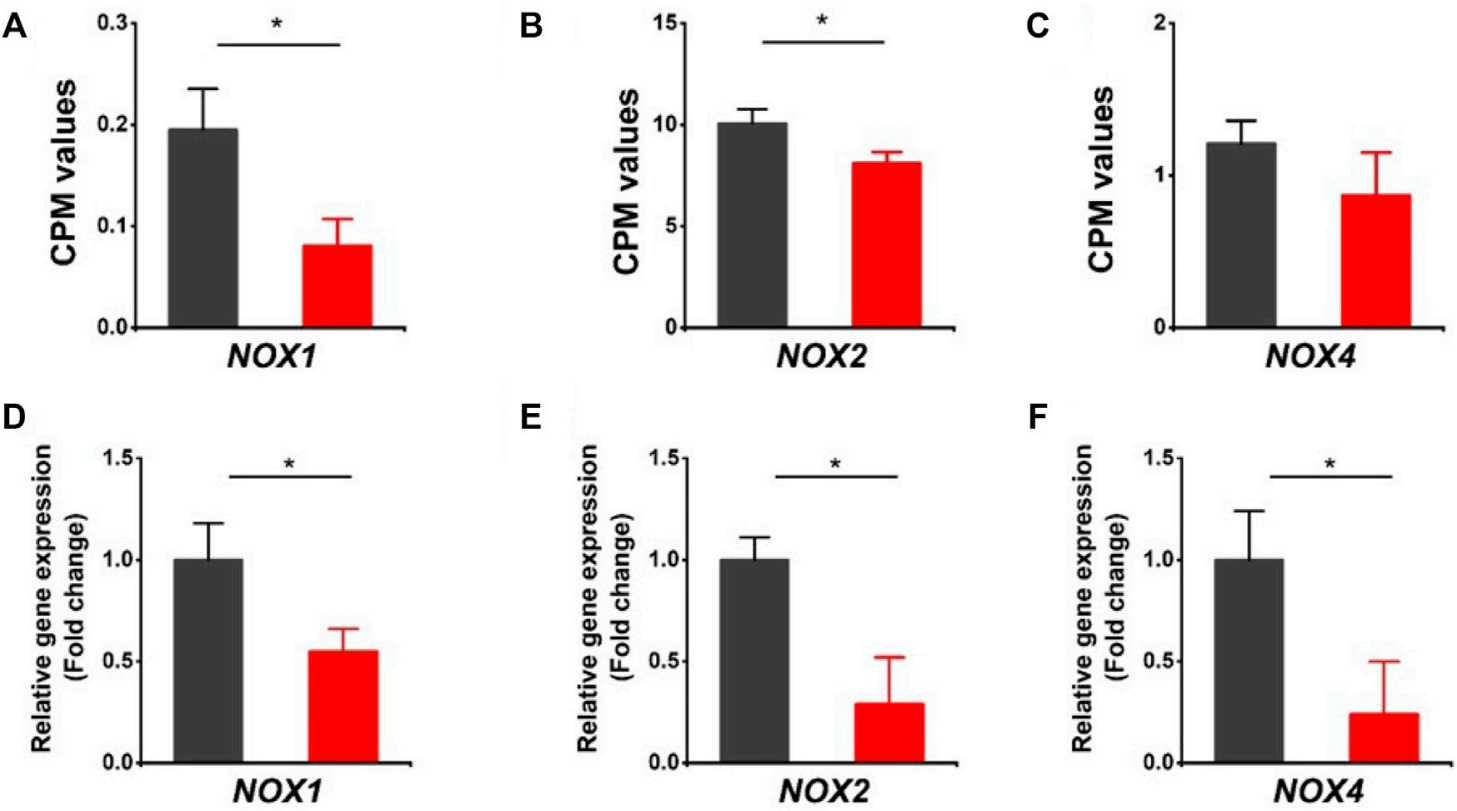
Downregulation of the NOX family of ROS-generating NADPH oxidases in HVCN1^−/−^ hearts. Comparing relative gene expression levels of *NOX1*, *NOX2*, and *NOX4* between WT (black) and HVCN1^−/−^ (red) detected by RNA-seq **(A-C)** and qPCR **(D-F)**. n = 4 independent experiments for RNA-seq; n = 3 independent experiments for qPCR; *p < 0.05.

It is noted that both oxidoreductase activity and oxidation-reduction process were downregulated in HVCN1 null hearts from the GO enrichment analysis of top significant gene groups (Figure 1D). The oxidoreductase utilizes NADP^+^ or NAD^+^ as cofactors to catalyze the transfer of electron between molecules, and HVCN1 provides charge and pH compensation during the NADPH oxidoreductases (NOXs) activation (Seredenina et al., 2015). Since HVCN1 was coupled with NOX activity, we determined the effects of HVCN1 deletion on the expression of NOX family. We detected three isoforms of the NOX family in the hearts, including *NOX1*, *NOX2*, and *NOX4* (Figure 2). The RNA-seq results showed that three isoforms were downregulated in HVCN1 null hearts (Figures 2A–C). RT-PCR confirmed changes in RNA-seq, and the mRNA expression levels of all three NOX isoforms were markedly reduced in the HVCN1^−/−^ hearts compared with the ones in the WT hearts (Figures 2D–F), indicating that deletion of HVCN1 was associated with gene expression of the NOX family.

### Differentially Expressed Genes Related to Cl^−^/HCO_3_^−^ Exchangers

**FIGURE 3:**
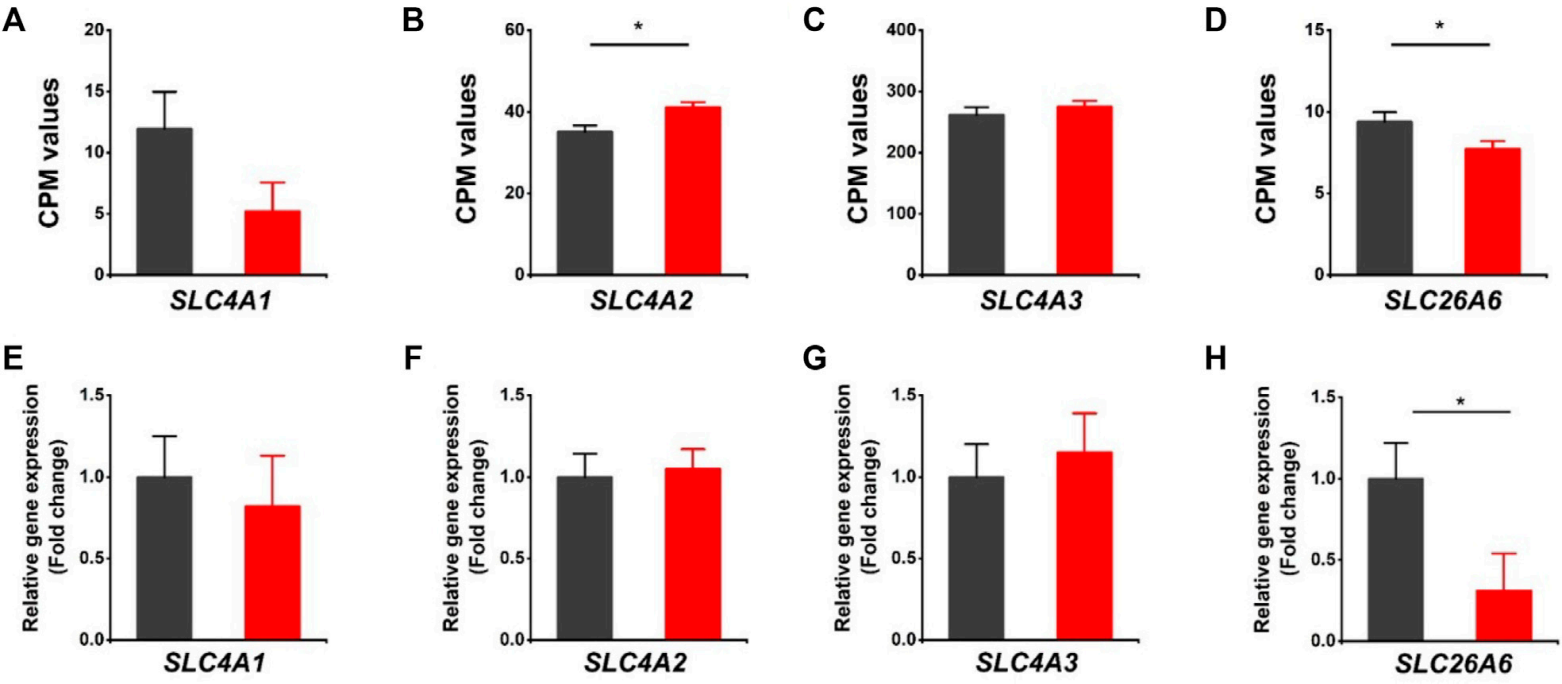
Differentially expressed genes related to Cl^−^/HCO3^-^ exchangers in HVCN1^−/−^ hearts. Comparing relative gene expression levels of *SLC4A1* (AE1), *SLC4A2* (AE2), *SLC4A3* (AE3), *SLC26A6* between WT (black) and HVCN1^−/−^ (red) detected by RNA-seq **(A-D)** and qPCR **(E-H)**. n = 4 independent experiments for RNA-seq; n = 3 independent experiments for qPCR; *p < 0.05.

The HVCN1 has been proposed to combine with HCO_3_^−^ exchangers in regulating transport-mediated CO_2_ disposal in the heart (Vairamani et al., 2017). We assessed the expression of bicarbonate transporters in the HVCN1 null hearts. The *SLC4A1* (encoding Anion Exchanger 1, AE1), *SLC4A2* (encoding Anion Exchanger 2, AE2), *SLC4A3* (encoding Anion Exchanger 3, AE3), and *SLC26A6* are the most abundant Cl^−^/HCO_3_^−^ exchangers expressed in hearts (Figures 3A–D). The RNA-profiling and RT-PCR results showed HVCN1^−/−^ significantly downregulated *SLC26A6* (Figures 3D–H), but did not significantly affect the AE1 and AE3 (Figures 3A,C,E, G). Although RNA-seq data indicated increased AE2 in HVCN1 null hearts (Figure 3B), the RT-PCR analysis showed no significant alterations of AE2 expression between groups (Figure 3F). These data suggested that HVCN1 might affect the expression of *SLC26A6* in the regulation of cardiac CO_2_ homeostasis.

### Differentially Expressed Genes Related to Other Proton Channels and Proton-Coupled Transporters/Antiporters

**FIGURE 4:**
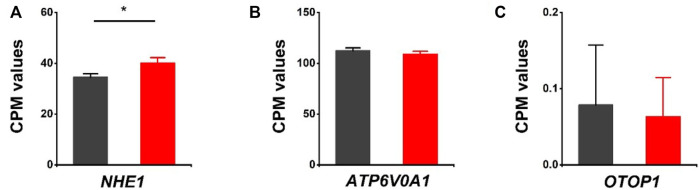
Differentially expressed genes related to other proton transporters/channels in HVCN1^−/−^ hearts. Comparing relative gene expression levels of proton transporters between WT (black) and HVCN1^−/−^ (red) including sodium-hydrogen exchanger 1 (*NHE1*) **(A)**, a1-subunit of the V_0_ domain of V-type proton ATPase (*ATP6V0A1*) **(B)**, and otopetrin proton channel 1 (*OTOP1*) **(C)**, n = 4 independent experiments for RNA-seq; *p < 0.05.

The effects of HVCN1 proton channel deletion on other proton channels/exchangers and proton-coupled transporters/antiporters were investigated. The sodium-hydrogen exchanger 1 (*NHE1*) has been reported to regulate intracellular pH in cardiomyocytes (Sundset et al., 2003). Our RNA-seq revealed that *NHE1* was upregulated in HVCN1 null hearts. The increased expression of *NHE1* might be a functional compensation for the H^+^ transport burden due to the deletion of the HVCN1 proton channel (Figure 4A). Additionally, proton pump (V-type proton ATPase, *ATP6V0A1*) and proton channel otopetrin 1 (*OTOP1*) were not altered in the HVCN1 null hearts (Figures 4B,C).

**FIGURE 5:**
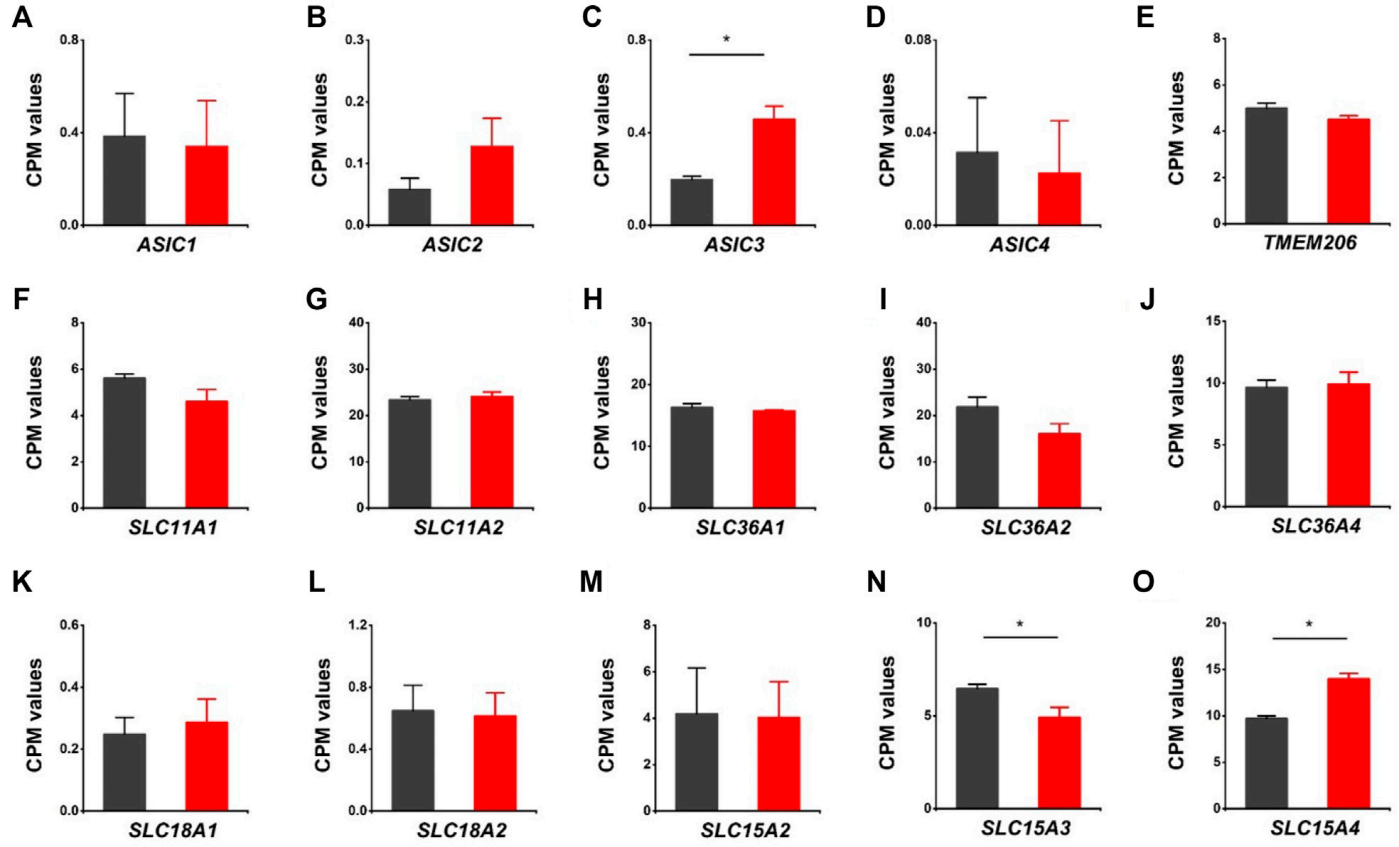
Differentially expressed genes related to proton-coupled channels/transporters/antiporters in HVCN1^−/−^ hearts. Comparing relative gene expression levels of proton-coupled transporters between WT (black) and HVCN1^−/−^ (red) including acid-sensing ion channels *ASIC1, ASIC2, ASIC3, ASIC4* **(A-D)**, proton activated chloride channel *TMEM206* **(E)**, proton-coupled metal-ion transporters *SLC11A1*, *SLC11A2* **(F-G)**, proton-coupled amino acid transporters *SLC36A1*, *SLC36A2*, *SLC36A4* **(H-J)**, proton/amine antiporters *SLC18A1*, *SLC18A2* **(K-L)**, and proton-dependent oligopeptide transporters *SLC15A2*, *SLC15A3*, *SLC15A4* **(M-O)**. n = 4 independent experiments for RNA-seq; *p < 0.05.

We further assessed the expression of acid-sensing ion channels (*ASIC1, ASIC2, ASIC3, ASIC4*), proton activated chloride channel (*TMEM206*), proton-coupled metal-ion transporters (*SLC11A1*, *SLC11A2*), proton-coupled amino acid transporters (*SLC36A1*, *SLC36A2*, *SLC36A4*), and proton/amine antiporters (*SLC18A1*, *SLC18A2*). Except for *ASIC3*, there were no notable changes in those genes associated with the HVCN1 deletion (Figures 5A–L). Moreover, the proton-dependent oligopeptide transporters showed differential expression linked with the HVCN1^−/−^. Although the expression of *SLC15A2* was not changed, the expression of *SLC15A3* was decreased, and *SLC15A4* was increased (Figures 5M–O), indicating a role of HVCN1 in differential regulation of proton-dependent oligopeptide transport.

### Differentially Expressed Genes Related to Cardiac Ion Channels in HVCN1^−/−^ hearts

**FIGURE 6:**
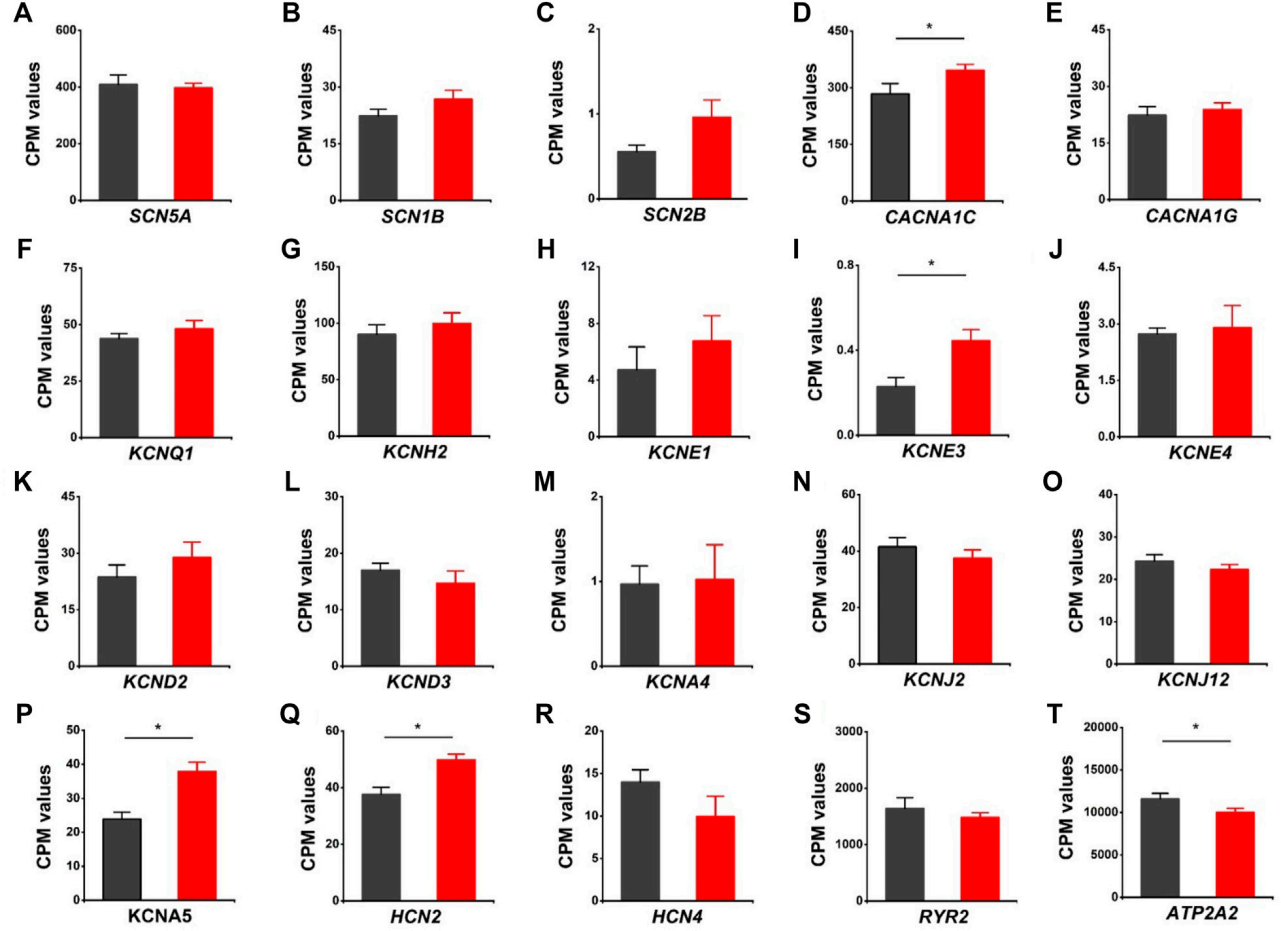
Differentially expressed genes related to cardiac ion channels in HVCN1^−/−^ hearts. Comparing relative gene expression levels of cardiac ion channels between WT (black) and HVCN1^−/−^ (red) including cardiac sodium channel *SCN5A* **(A)**, sodium channel beta subunits *SCN1B* and *SCN2B* **(B-C),** cardiac L-type calcium channel *CACNA1C* **(D)**, T-type calcium channel *CACNA1G* **(E)**, cardiac potassium channels *KCNQ1* **(F)**, *KCNH2* **(G)**, potassium channel beta subunits *KCNE1* **,** *KCNE3* **,** *KCNE4* **(H-J),** I_to_ channels *KNCD2*, *KCND3*, *KCNA4* **(K-M)**, I_K1_ channels *KCNJ2*, *KCNJ12* **(N-O)**, I_kur_ channel *KCNA5* **(P)**, I_f_ channels *HCN2*, *HCN4* **(Q-R)**, and cardiac ryanodine receptor *RYR2* **(S)** and SERCA pump *ATPA2* **(T)**. n = 4 independent experiments for RNA-seq; *p < 0.05.

We evaluated the effects of HVCN1 deletion on the expression profiles of cardiac ion channels, including cardiac sodium channel alpha subunit (*SCN5A*), sodium channel beta subunits *SCN1B* and *SCN2B*, L-type and T-type calcium channels (*CACNA1C*, *CNCNA1G*), I_Ks_, I_Kr_, I_to_, I_K1_, I_Kur_ potassium channels (*KCNQ1, KCNH2, KCNE1, KCNE3, KCNE4, KCND2, KCND3, KCNA4, KCNJ2, KCNJ12, KCNA5*), I_f_ channels (*HCN2*, *HCN4*), and sarcoplasmic reticulum calcium handling proteins (*RYR2*, SERCA pump) (Figure 6). The knockout hearts exhibited upregulation of L-type calcium channel *CACNA1C*, potassium channel beta subunit *KCNE3*, I_Kur_ potassium channel *KCNA5*, I_f_ channel *HCN2* (Figures 6D,I, P, Q), and downregulation of SERCA pump *ATPA2* (Figure 6T). The data supported that HVCN1 was involved in the regulation of cardiac electrophysiology, and HVCN1 deletion potentially remodeled action potential profiles in cardiomyocytes.

## Discussion

The present study suggested that HVCN1 regulated gene transcriptional networks controlling NOX signaling and CO_2_ homeostasis in the heart. The RNA-profiling data indicating impaired pH homeostasis in the HVCN1^−/−^ hearts were the downregulated NADPH oxidoreductases (NOXs), decreased expression of Cl^−^/HCO_3_^−^ exchanger *SLC26A6*, and differential expression of cardiac ion transporters/channels.

HVCN1 channel regulates NOX-mediated respiratory burst to control the production of ROS/H_2_O_2_ in the cells. The NOX family has seven members, including NOX1, NOX2, NOX3, NOX4, NOX5, Duox1, and Duox2 (Bedard et al., 2015). During the respiratory burst, NOX uses NADPH as an electron donor and molecular oxygen as an electron acceptor to produce O_2_^−^. This activity leaves excess proton (H^+^) in the cytoplasm. Proton extrusion by the HVCN1 channel compensates for the electrogenic activity of NOX and limits the inhibition of the enzyme induced by cytoplasmic acidification, thus favoring the efficient generation of ROS/H_2_O_2_ from O_2_ (Seredenina et al., 2015).

Three isoforms, including NOX1, NOX2, and NOX4, were detected in mouse hearts. We showed that HVCN1 deletion significantly influenced the expression of the NOX family, and mRNA expression levels of all NOX isoforms were reduced in the HVCN1^−/−^ hearts. The downregulated NOXs likely generate disturbance in the production of H_2_O_2_. The NOX-medicated ROS signaling pathways regulate heart diseases’ physiological or pathophysiological processes (Pena et al., 2020; Zhang et al., 2020). It was reported that NOX2 activity was associated with angiotensin II-induced myocyte hypertrophy (Hingtgen et al., 2006), and NOX1 mediated myocyte death under stress situations (Matsuno et al., 2012). Moreover, high-level expression of NOX4 was shown to play essential roles in hypoxia and heart failure (Kuroda et al., 2010; Zhang et al., 2010). Our results suggested a potential role of the HVCN1 channel in heart diseases, such as hypertrophy and heart failure associated with NOX-medicated ROS signaling pathways. In pathophysiological conditions, inflammation and excessive stress cause cardiac dysfunction. As compensation, myocyte cells generate more ROS to activate cellular signaling pathways to counteract the abnormal stress and stimulation. In the long run, the activation of these pathways leads to myocardial hypertrophy *via* H_2_O_2_ (Zafari et al., 1998), which is the primary ROS regulated by HVCN1.

**FIGURE 7:**
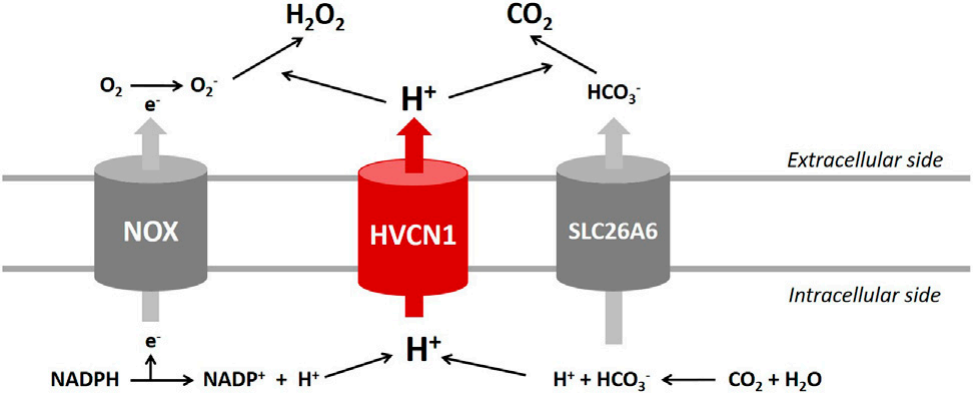
Proposed roles of HVCN1 in regulating pH homeostasis in the heart. Proton (H^+^) extrusion by the HVCN1 channel compensates for the electrogenic activity of NOX and regulates the efficient generation of H_2_O_2_. In addition, HVCN1-mediated H^+^ current is associated with transport-mediated CO_2_ disposal. Except for HVCN1 functional regulation of pH homeostasis, the present study revealed that HVCN1 regulated the genetic expression of *NOXs* and *SLC26A6* in the heart.

The HVCN1 channel was implicated in HCO_3_^−^ transport in lung epithelium and regulated airway surface liquid pH (Fischer, 2012). Studies reported that HCO_3_^−^ and HCO_3_^−^-handling proteins played essential roles in regulating cardiac function (Wang et al., 2014). Recent transcriptome analyses indicated that the voltage-gated proton channel HVCN1 mRNAs were expressed in the heart at high levels comparable to those of Cl^−^/HCO_3_^−^ exchangers (Brawand et al., 2011; Yu et al., 2014; Vairamani et al., 2017), and Cl^−^/HCO_3_^−^ exchanger AE3 was proposed to combine with HVCN1-mediated H^+^ currents to generate transport-mediated CO_2_ disposal in the heart (Vairamani et al., 2017). The present study revealed that HVCN1^−/−^ significantly downregulated *SLC26A6* but did not influence the expression of AE3. We speculate that HVCN1 could alter the expression of *SLC26A6* regulating cardiac CO_2_ homeostasis (Figure 7).

Additionally, the RNA-seq results provided limited support that HVCN1 was involved in modulating cardiac electrophysiology. The HVCN1 knockout hearts exhibited differential expression of cardiac ion channels, including upregulated L-type calcium channel, I_Kur_, HCN2, and downregulated SERCA pump. These results indicated that HVCN1 was associated with the electrophysiological remodeling in cardiomyocytes, and HVCN1 might regulate cardiac ion channels function like other channel partners (Hong et al., 2020; Wu and Hong, 2021). Previous studies reported that deficits of the HVCN1 channel by the gene deletion or pharmacological block produced intracellular acidification (low pH_i_) in a variety of cell types (Cherny and DeCoursey, 1999; Wu et al., 2012; Asuaje et al., 2017). The deletion of HVCN1 likely also alters the intracellular pH in myocytes. It was known that cellular pH was an essential modulator of cardiac function, and low pH_i_ regulated many cardiac ion channel functions (Vaughan-Jones et al., 2009). The low pH_i_ reduced transient outward K^+^ currents (I_to_) current amplitude (Saegusa et al., 2013), stimulated L-type calcium channel (I_Ca,L_) gating process (Saegusa et al., 2011), and weakened rectification of the cardiac K_ATP_ channel (Baukrowitz et al., 1999). Through effects on the Ca^2+^ and K^+^ conductance pathways, low pH_i_ modified action potential (AP) profiles and induced cardiac arrhythmias. Moreover, the low pH_i_-induced Ca^2+^-overload could result in mitochondrial dysfunction and defects in contractility, accounting for cardiac hypertrophy and heart failure (Duchen et al., 2008). However, the effects of HVCN1-mediated pH homeostasis on the cardiac ion channel function remain unclear. Studies in the field will help explore the role of HVCN1 in cardiac electrophysiology.

In summary, the present study highlights the importance of HVCN1 in cardiac function and may present a novel target associated with heart diseases. The RNA-seq data indicated that HVCN1 was involved in cardiac pH homeostasis (Figure 7) and showed that HVCN1 regulated gene transcriptional networks controlling NOX signaling and CO_2_ homeostasis, suggesting a potential role of the HVCN1 channel in heart failure and cardiomyopathy, which are associated with abnormal expression of NOX and bicarbonate transporters. Additionally, HVCN1 dysfunction caused intracellular acidification in various cells. As the cellular pH has a crucial influence on cardiac contractility and rhythm, the pH disturbances causing reversible contractile dysfunction are linked with cardiac arrhythmias. Future functional studies on the effects of HVCN1 on cardiac electrophysiology are required to address these uncertainties.

## Data Availability

The datasets presented in this study can be found in online repositories. The names of the repository/repositories and accession number(s) can be found below: GEO, GSE195945.
